# *In Vitro* and *In Vivo* Antitumor Activity of Cucurbitacin C, a Novel Natural Product From Cucumber

**DOI:** 10.3389/fphar.2019.01287

**Published:** 2019-11-08

**Authors:** Dinglan Wu, Zhu Wang, Muqi Lin, Yi Shang, Fei Wang, JiaYi Zhou, Fei Wang, Xiantong Zhang, Xiaomin Luo, Weiren Huang

**Affiliations:** ^1^Shenzhen Key Laboratory of Viral Oncology, The Clinical Innovation & Research Centre, Shenzhen Hospital, Southern Medical University, Shenzhen, China; ^2^Department of Urology, People’s Hospital of Longhua Shenzhen, Southern Medical University, Shenzhen, China; ^3^School of Pharmaceutical Sciences, Health Science Center, Shenzhen University, Shenzhen, China; ^4^Agricultural Genome Institute at Shenzhen, Chinese Academy of Agricultural Science, Shenzhen, China; ^5^Department of Urology, The Hospital of Hainan Province, Haikou, China; ^6^Department of Urology, Shenzhen Second People’s Hospital, The First Affiliated Hospital of Shenzhen University, International Cancer Center, Shenzhen University School of Medicine, Shenzhen, China

**Keywords:** cucurbitacin C, natural product, anti-cancer, growth arrest, apoptosis, Akt pathway

## Abstract

Cucurbitacin C (CuC), a novel analogue of triterpenoids cucurbitacins, confers a bitter taste in cucumber. Genes and signaling pathways responsive for biosynthesis of CuC have been identified in the recent years. In the present study, we explored the anti-cancer effects of CuC against human cancers *in vitro* and *in vivo*. CuC inhibited proliferation and clonogenic potential of multiple cancer cells in a dose-dependent manner. Low-dose CuC treatment induced cell cycle arrest at G1 or G2/M stage in different cancer lines, whereas high-dose treatment of CuC caused apoptosis in cancer cells. PI3K-Akt signaling pathway was found to be one of the major pathways involved in CuC-induced cell growth arrest and apoptosis by RNA-Seq and Western blotting. Mechanistic dissection further confirmed that CuC effectively inhibited the Akt signaling by inhibition of Akt phosphorylation at Ser473. *In vivo* CuC treatment (0.1 mg/kg body weight) effectively inhibited growth of cancer cell-derived xenograft tumors in athymic nude mice and caused significant apoptosis. Our findings for the first time demonstrated the potential therapeutic significance of CuC against human cancers.

## Background

Cucurbitacins are triterpenoid molecules originally isolated as bitter principles from Cucurbitaceae plants. So far, 12 categories of cucurbitacins have been identified based on molecular structural differences and classified as cucurbitacins A–T (Chen et al., 2005; Kaushik et al., 2015). Cucurbitacins have been gaining attention of scientists for their potential anti-cancer effects for decades (Lee et al., 2010; Alghasham, 2013; Cai et al., 2015), which process a broad range of pharmacological activities, including anti-inflammatory, antipyretic, and anti-cancer activities (Kaushik et al., 2015). In particular, the anti-cancer activities of cucurbitacins through antiproliferation (Mao et al., 2019; Saeed et al., 2019), inhibition of migration and invasion (Touihri-Barakati et al., 2017; Zou et al., 2018), induction of cell apoptosis (Ding et al., 2017; He et al., 2017; Liu et al., 2018; Mao et al., 2019), autophagy (Ni et al., 2018; Lin et al., 2019), and cell cycle arrest promotion are of great interest (Liu et al., 2018). For instance, cucurbitacins B, D, E, and I are the most wildly studied variants and exhibit general *in vitro* and *in vivo* anti-cancer effects. Cucurbitacin B shows significant inhibition effects in breast cancer (Jin et al., 2018), liver cancer (Ge et al., 2018), and glioblastoma multiforme (Qin et al., 2018); cucurbitacin E exerts anti-cancer activities in treating brain cancer (Cheng et al., 2019) and gastric cancer (Jafargholizadeh et al., 2018); cucurbitacin D is effective to treat cervical cancer (Sikander et al., 2016) and breast cancer (Ku et al., 2015); and cucurbitacin I also processes anti-cancer activities (Kim and Kim, 2015; Wu et al., 2016; Ni et al., 2018). Moreover, cucurbitacins can also inhibit tumor angiogenesis (Touihri-Barakati et al., 2017; Piao et al., 2018), enhance anti-proliferative activity of chemotherapy drugs (Sadzuka et al., 2010; Aribi et al., 2013), and suppress cancer cell stemness (Shukla et al., 2016).

Cucurbitacin C (CuC), one of the variants of cucurbitacin identified in 1954 as a bitter substance only being found in cucumber (*Cucumis sativus* L.), exists in both the leaves and fruits of the cucumber plant (Qing et al., 2014). A recent study dissected the gene networks of CuC biosynthetic pathway, which was controlled by two transcriptional factors, *Bi* (Bitter leaf) and *Bt* (Bitter fruit) (Shang et al., 2014). CuC was produced as a natural toxic repellent that can effectively kill, repel, or weaken the attacking organisms. Given the promising anti-cancer activities of the cucurbitacin analogues, CuC might be a significant candidate for drugs. However, no report has been published on the pharmacological activities and anti-cancer effects of CuC, and the potential signaling pathways had never been investigated.

In the present study, we sought to demonstrate if the CuC could act as a novel anti-tumor agent in human common malignancies, such as prostate cancer, bladder cancer, and liver cancer. We showed for the first time that the natural product CuC inhibited cancer cell growth *in vitro* and *in vivo* by induction of growth arrest, cellular migration inhibition, and apoptosis in several types of cancer cells. Moreover, results from a molecular mechanism study showed that CuC attenuates Akt pathway to mediate its anti-cancer activities.

## Materials and Methods

### Cell Lines

Human lung cancer cell A549; colon cancer cell HCT116; bladder cancer cell T24; human prostate cancer cell lines LNCaP, DU145, and PC-3; and hepatoblastoma-derived cell line HepG2 (Lopez-Terrada et al., 2009) were obtained from American Type Culture Collection (ATCC). T24 and HepG2 cells were cultured in DMEM. LNCaP was kept in RPMI-1640 medium, DU145 in MEM, A549 and PC-3 in F12K medium, and HCT116 in McCoy’s 5a medium. All cultured media were supplement with 10% fetal bovine serum (FBS), 100 units/ml penicillin sodium, and 100 μg/ml streptomycin as described previously (Wang et al., 2018). Cultures were maintained in a 5% CO_2_ humidified atmosphere at 37°C.

### Reagent and Antibodies

The isolation of CuC from leaves and fruits of cucumber has been described previously (Qing et al., 2014). In short, green leaves or fruits were soaked in 95% alcohol at room temperature, then evaporated and separated on silica gel column eluted with chloroform and methanol, and further purified by semi-preparative high-performance liquid chromatography (HPLC) system. The purity of CuC was detected by liquid chromatography–mass spectrometry (LC-MS) analysis (**Supplementary Figure 2**). The compounds were prepared as a 20 mM stock solution in DMSO. The stock solutions were stored in aliquots at −20°C and diluted with culture medium.

The following antibodies were used in this study: cyclin A, cyclin D1, p21, p27, and p53 (DO-1) were bought from Santa Cruz; Apoptosis Antibody Sampler Kit (9915T), bcl-2, caspas-8, cleaved caspas-8, Akt, p-Akt, β-actin, and GAPDH were bought from Cell Signaling Technology.

### Cell Viability Assay

Cell viability was determined with a 3-(4,5-dimethylthiazole-2-yl)-2,5-biphenyl tetrazolium bromide (MTT) assay. Cells at 70∼80% confluence were typsined and plated into a 96-well plate at 2∼5 × 10^3^/well. After an overnight incubation, cells were treated with various concentrations (1 nM–10 μM) of CuC (log or half-log dilution) for 48 h. The percentage of survival cells was calculated as the ratio (A570) of treated cells over control cells. The final data represented mean ± standard deviation (SD) of three independent experiments.

### Colony Formation Assay

Cells were suspended in culture medium and plated at 500 cells/well onto six-well plates and treated with various concentrations of CuC. After 14 days of culture, cells were fixed in 4% paraformaldehyde and stained with 0.1% crystal violet. Stained colonies with sizes larger than 100 mm were counted under a dissecting microscope to determine the clonal formation efficiency (percentage of colonies formed from 500 seeded cells).

### Would Healing Assay

Cells were cultured in 6-well plates at 1 × 10^5^ cells/well to confluent monolayers, which then were starved in serum-free medium overnight. Straight wounds were made by using 200-µl pipette tips. After washing with medium to remove cell debris, wounded monolayers were incubated in medium with 1% FBS to minimize cell proliferation and treated with 20 nM CuC or vehicle. The wound gaps were photographed at regular intervals (0, 12, and 24 h), and the area of cell-free wounds was measured using morphometry software (Motic Images 2.0). Results of assays were obtained from at least three independent experiments.

### Flow Cytometry for Cell Cycle and Apoptosis Analysis

Cells at 80% confluence were treated with CuC in complete medium for 48 h and harvested. For cell cycle assay, cells were resuspended in phosphate-buffered saline (PBS) (1 × 10^6^/ml) and fixed in ice-cold 70% ethanol overnight at −20°C. Fixed cells were incubated in staining buffer with 50 μg/ml propidium iodide (PI) and 10 μg/ml DNase-free RNase for 15 min; 3 × 10^5^ cells were used to do the DNA flow cytometry (FACS Calibur BD Flow Cytometer, Sony SA3800 Spectral Analyzer). For apoptosis assay, the cells were rinsed with PBS and resuspend in annexin V-FITC and PI buffer for 15 min in dark. The stained apoptotic cells were counted by a FACS Calibur Flow Cytometer system. For each sample, data from 10,000 cells were recorded.

### Western Blot Analysis

The treated and control cells were rinsed with PBS and lysed on ice with lysis buffer (50 mM Tris [pH 7.4], 50 mM NaCl, 1 mM DTT, 1 mM sodium orthovanadate, 1 mM EDTA, 1 mM EGTA, 0.5% Sodium dodecyl sulfate [SDS], and 1% NP40, with protease inhibitors cocktail [Roche]) and placed on ice for 15 min. The insoluble protein lysate and DNA were cleaned by centrifugation at 12,000 rpm for 5 min at 4°C. Protein concentrations were quantified using the Bio-Rad protein Assay kit. Whole-cell lysates (20∼40 μg) were resolved by 8∼15% SDS–polyacrylamide gel electrophoresis, transferred to a polyvinylidene difluoride membrane, and subjected to Western blot analysis with the specific antibodies. The signal was visualized with the Enhanced Chemi-luminescence Plus (ECL Plus) detection system (GE Healthcare).

### RNA-Seq and Bioinformatics Analysis

HepG2 and PC-3 cells were treated with or without CuC for 24 h, with each experiment repeated twice. Total RNA was extracted using RNeasy kit (Qiagen), and RNA concentration was quantified. The quality of total RNA was analyzed using Agilent 2100. Sequencing libraries construction and paired-end deep sequencing were performed by Novogene (Tianjin, China) using Illumina PE150 system according to the manufacturers’ instructions. Gene expression values were further used to calculate for library size and data set dispersion for differentially expressed gene analysis. Gene set functional and pathway analysis were analyzed using Gene Ontology (GO) and Kyoto Encyclopedia of Genes and Genomes (KEGG) pathway.

### Xenograft Mouse Models

HepG2 (3 × 10^6^) or PC-3 cells were subcutaneously inoculated into the flank region of 7-week-old male severe combined immunodeficiency (SCID) mice and allowed to grow for 3 weeks for HepG2 and 8 weeks for PC-3. Tumor growth was monitored weekly, and tumor volumes (mm^3^) were measured by electronic calipers, using the following formula: *V* = (*W*^2^ × *L*)/2 (*L* is the longest diameter). When the tumor volume reached ∼200 mm^3^, mice were treated with CuC (0.1 mg/kg) and the respective vehicle control (1× PBS) by intraperitoneal (i.p.) injection three times per week for 4 weeks. Body weight and the tumors were regularly monitored. At the end of experiment, mice were sacrificed by non-painful anesthesia, and their tumors were fixed and paraffin-embedded. All animal protocols were approved by the Animal Experimentation Ethics Committee, Southern Medical University.

### TUNEL Assay

We applied the TdT-mediated dUTP-X nick end labeling (TUNEL) test using the *in situ* cell death detection kit and fluorescein (Roche) to detect DNA double-strand breaks induced by CuC. Briefly, paraffin sections of HepG2 and PC-3 xenografts were dewaxed and rehydrated according to standard protocols by heating at 60°C followed by washing in xylene and rehydration through a graded series of ethanol and double distilled water. After being incubated with permeabilization solution (0.1% Triton X-100, 0.1% sodium citrate) for 8 h and rinsed twice with PBS, slides were added with 50 μl TUNEL reaction mixture in a humidified atmosphere for 60 min at 37°C in dark. Slides then will be washed three times in PBS and mounted with SlowFade Gold antifade (Life Technologies) with Hoechst 33342. Fluorescence images were acquired using the fluorescence imaging system (Olympus).

### Statistics Analysis

Statistical analyses were performed using GraphPad Prism 5 software (GraphPad Software). All results are presented as mean ± SD from at least three independent experiments. Student’s *t*-tests were performed to analyze the differences between two groups. ANOVA tests and Dunnett’s test were used for comparisons between multiple groups. Differences were considered statistically significant where *P* values < 0.05.

## Results

### CuC Inhibits the Proliferation and Clonogenic Potential of Prostate, Bladder, and Liver Cancer Cells *InVitro*

**Figure 1:**
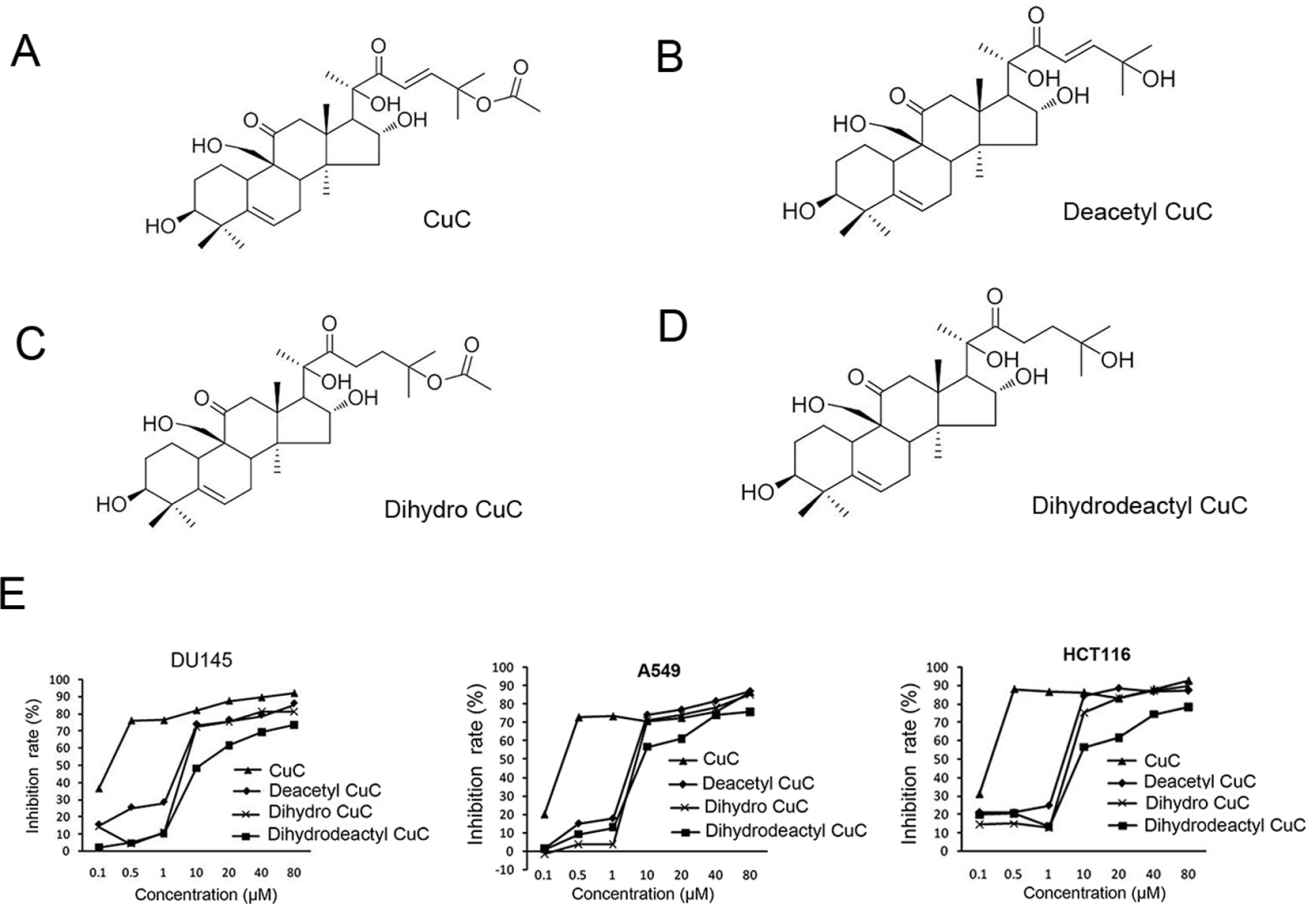
Molecular structure of CuC and its derivatives. **(A)** CuC. **(B)** Deacetyl CuC. **(C)** Dihydro CuC. **(D)** Dihydrodeactyl CuC. **(E)** *In vitro* growth response of prostate cancer cell line DU145, human lung cancer cell line A549, and colon cancer cell HCT116 upon treatment of CuC and its derivatives. CuC shows the highest inhibition rate.

**Figure 2:**
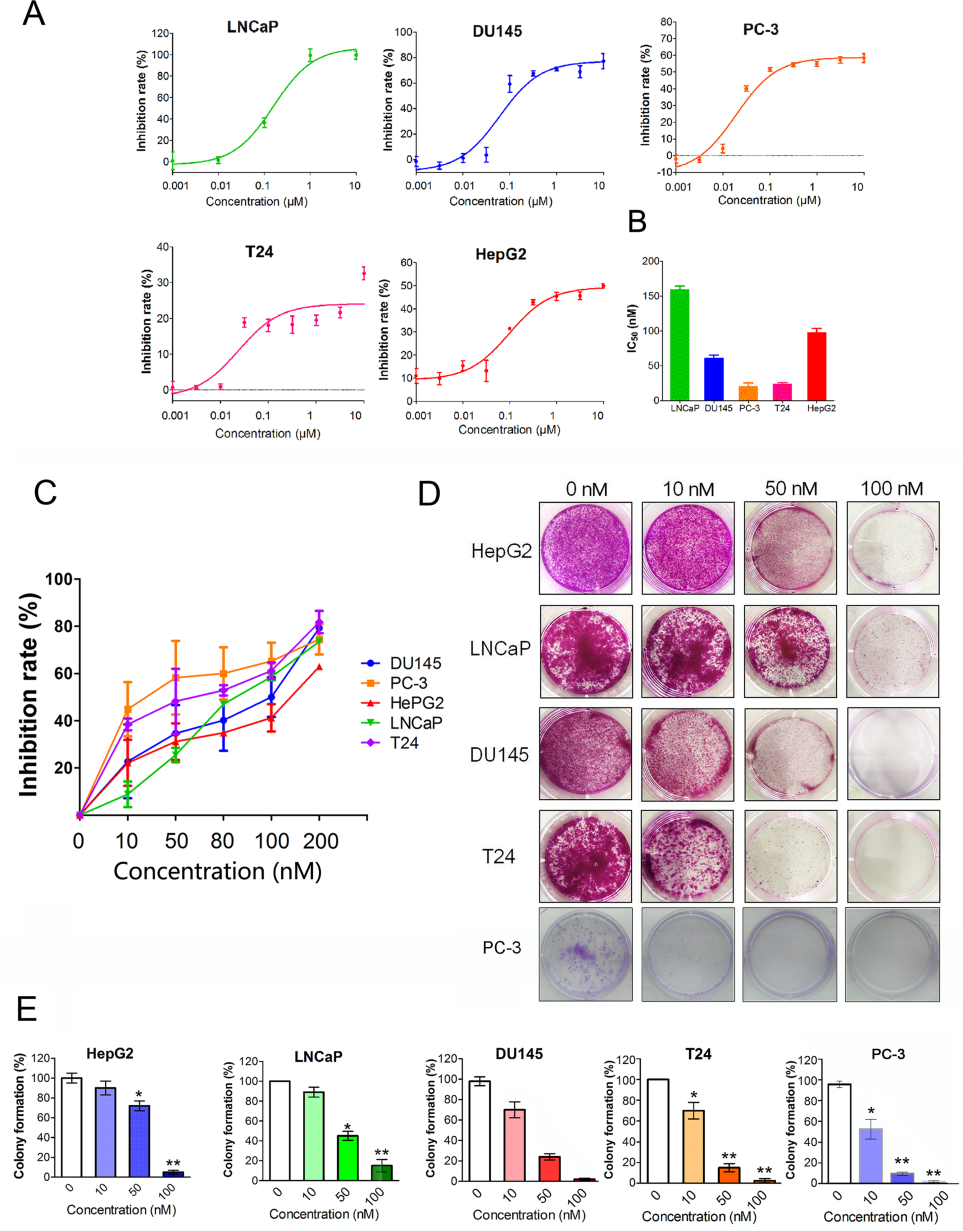
CuC inhibits cancer cell growth. **(A)** *In vitro* growth response of prostate cancer cell line (LNCaP, DU145, and PC-3), bladder cancer cell line T24, and hepatoblastoma-derived cell line (HepG2) upon CuC treatment. Results showed that CuC dose dependently inhibits the growth of all cancer cell lines. **(B)** IC50 of CuC treatment in cancer lines. **(C)** Inhibition rates of CuC treatment (0–200 nM) in cancer lines. PC-3 is the most sensitive cancer line upon CuC treatment. **(D)** Represent photos showing colony formation of several indicated cancer cells upon different dosages of CuC treatment for two weeks. **(E)** Statistic data show the colony formation efficiency of DU145 and HepG2 with CuC treatment, and results show that CuC significantly reduced the number of colonies (*, *P* < 0.05, **, *P* < 0.01).

**Figure 1** shows the chemical structure of CuC and its derivatives deacetyl CuC, dihydro CuC, and dihydrodeactyl CuC, which were extracted from leaves and fruits of cucumbers, and our data show that CuC displays the highest inhibition rates compared with its derivatives against cancer cells such as prostate cancer DU145, lung cancer cell A549, and colon cancer cell HCT116 (**Figure 1E**). To further determine the effect of CuC treatment on proliferation of cancer cells, MTT assay was performed on three prostate cancer cell lines (LNCaP, PC-3, and DU145), one bladder cancer cell line (T24), and one hepatoblastoma-derived cell line (HepG2) *in vitro* with a two-day exposure to serious concentration of CuC (0.001 μM–10 μM). We observed that CuC treatment dose dependently inhibited survival of cancer cells. All of these cancer lines showed significant sensitivity on CuC treatment (**Figure 2A**), and their IC50 ranged from 19.6 (PC-3) to 158.7 nM (LNCaP) (**Figure 2B**). A 0–200-nM serial dilution of CuC was also used to determine the optimal concentrations for CuC inhibition, and the results showed that 40%–60% inhibition rates distributed in the range of 10–100 nM CuC concentrations and PC-3 and T24 exhibited the highest inhibition rates among these cancer lines, which were consistent with their IC50 patterns (**Figure 2C**). We next performed colony formation assays to investigate the long-term treatment effect of CuC on proliferation of cancer cells. In this experiment, prostate cancer LNCaP, DU145, and PC-3 cells; hepatoblastoma HepG2 cells; and bladder cancer T24 cells were treated with CuC at nanomolar concentrations (10–100 nM) for two weeks. Results showed that CuC treatment significantly reduced the number of colonies formed in all cancer lines (**Figures 2D, E**) compared with their controls (*P* < 0.05). These findings suggested that CuC treatment inhibits the proliferation and clonogenic potential of cancer cells.

### CuC Induced Cell Cycle Arrest in Cancer Cells

**Figure 3:**
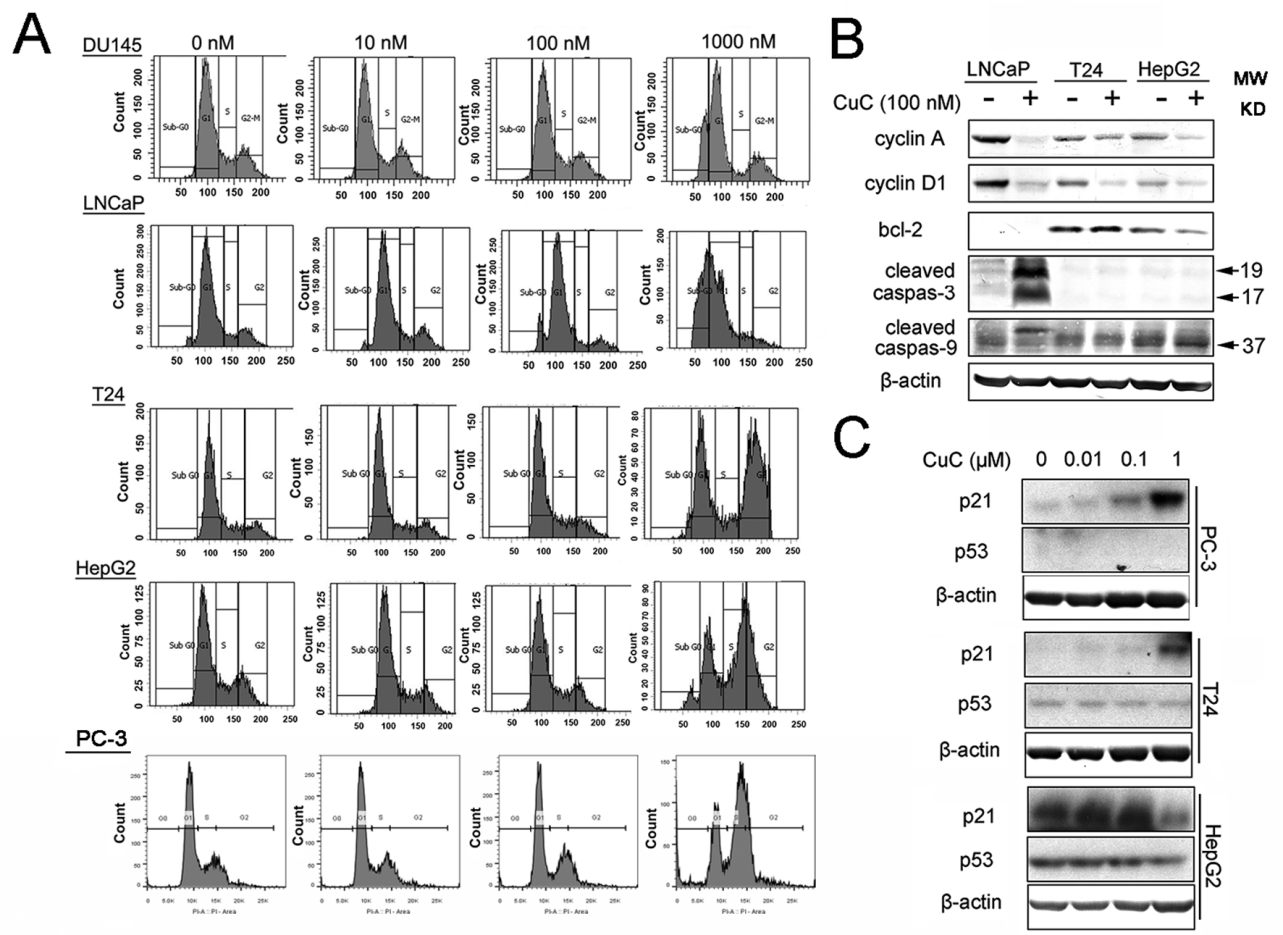
CuC induces cell growth arrest. **(A)** Flow cytometry analysis of the cell cycle phase of several indicated cancer cells subjected to CuC treatment for 48 h. The results show that serial concentration of CuC induced cell cycle arrest at G1 stage of DU145 and LNCaP cells but at G2/M in T24 cells, HepG2 cells, and PC-3 cells. **(B)** Western blot (WB) analysis of cycle-related proteins cyclin A and cyclin D1 and apoptosis-related proteins bcl-2, cleaved caspase-3, and cleaved caspase-9 in LNCaP, T24, and HepG2 were detected in the presence of 100 nM CuC or not. **(C)** WB analysis of p53 and p21 in PC-3, T24, and HepG2 cells after 24 h’ administration with serial concentration of CuC.

Induction of cell cycle arrest or apoptosis has been appreciated as a major target for cancer treatment. To investigate the inhibitory effect of CuC on cancer cell growth, cell cycle analysis was performed by flow cytometry. Cancer cells treated with a serial concentration of CuC for 48 h were subjected to DNA pattern analysis. Results showed that CuC treatment resulted in a significant blockage of DU145 and LNCaP cells at G1 stage but G2/M arrest in T24, HepG2 cells and PC-3 cells, when compared with vehicle treated group (**Figure 3A**). Moreover, a dramatic increase of cell population in sub-G1 fraction was induced by 1 μM CuC treatment, suggesting that the anti-proliferative activity of CuC also resulted from apoptosis induction. The DNA histograms were representatives of three independent experiments (**Figure 3A**).

To further understand the molecular mechanism underlying the effect of CuC on cell cycle arrest, we examined cell cycle and apoptosis regulatory proteins in cancer cells. Western blot analysis demonstrated that cyclin A and cyclin D1 were significantly decreased upon CuC treatment in LNCaP, T24, and HepG2 cells (**Figure 3B**). We next examined the effect of CuC on cell cycle inhibitory proteins p21 and p53. Western blot results showed that p21 was dose dependently up-regulated upon CuC treatment in PC-3, T24, and HepG2 cells. For PC-3 cells, p21 was highly accumulated at 1 μM treatment, while for HepG2 cells, in which the endogenous p21 was higher, this increase peaked at 0.1 μM dosage but down-regulated at 1 μM treatment. For all these three lines, p53 expression was not changed in either p53 wild type (HepG2) or p53 mutant (T24) or p53 lost cells (PC-3) (**Figure 3C**), indicating CuC-inducted p21 is p53-independent.

### CuC Induced Apoptosis in Cancer Cells

**Figure 4:**
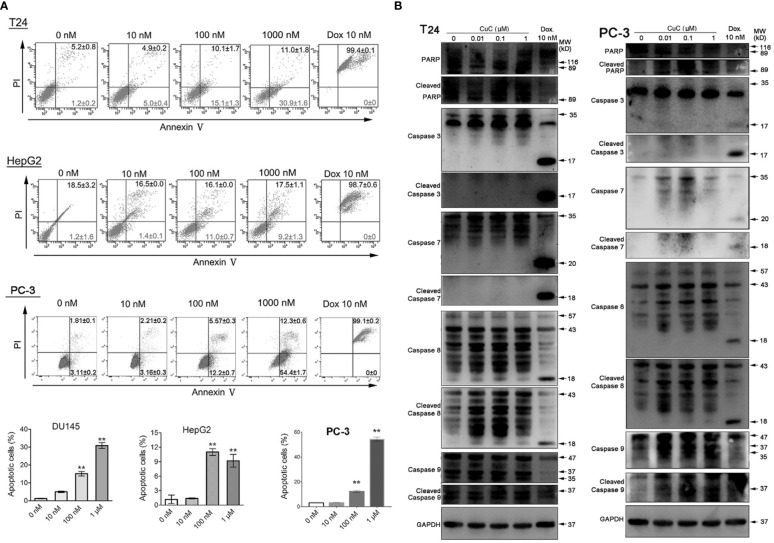
CuC induces cell apoptosis. **(A)** Apoptosis assay of cancer cells with CuC treatment by annexin V-FITC and PI staining. CuC induced significant early apoptosis at 100 mM and 1 µM treatment in T24 and HepG2 cells (**, *p* < 0.01). **(B)** Western blot (WB) analysis of indicated apoptotic markers after 48 h dosage of serial CuC in T24 and PC-3 cells.

Given that the anti-proliferative activity of CuC also resulted from apoptosis induction, to further elaborate our findings regarding the apoptosis induction of CuC on cancer, we performed apoptosis assay by flow cytometry using annexin V-FITC and PI staining method. Doxorubicin (Dox), an apoptosis inducer through direct oxidative DNA damage, was used as a positive control in this experiment. CuC treatment of T24, HepG2, and PC-3 cells for 24 h resulted in a dose-dependent increase of early apoptosis compared to control cells, whereas Dox significantly induced late apoptosis and cell death at 10 nM treatment (**Figure 4A**).

We further examined PARP, caspase-7/-8/-9, and their cleaved fragments by Western blot in PC3 and T24 cells under serial dosage treatment of CuC. Results showed that CuC dose dependently activated caspase-8 and PARP in both PC3 and T24 cells and could also activated initiator caspase-9 in PC-3 cells. The downstream effector elevated caspases-3/-7 had not be activated in T24 cells but slightly elevated in PC-3 cells (**Figure 4B**). Among the apoptosis-related markers, cleaved caspase-3 was significantly accumulated in LNCaP cells, and cleaved caspase-9 was increased in HepG2 cells. However, no significant change of bcl-2 level was observed in these cell lines (**Figure 3B**), indicating that induction of apoptosis by CuC in different cancer types is mediated through different pathways.

### CuC Inhibits Cell Migration of Cancer Cells

**Figure 5:**
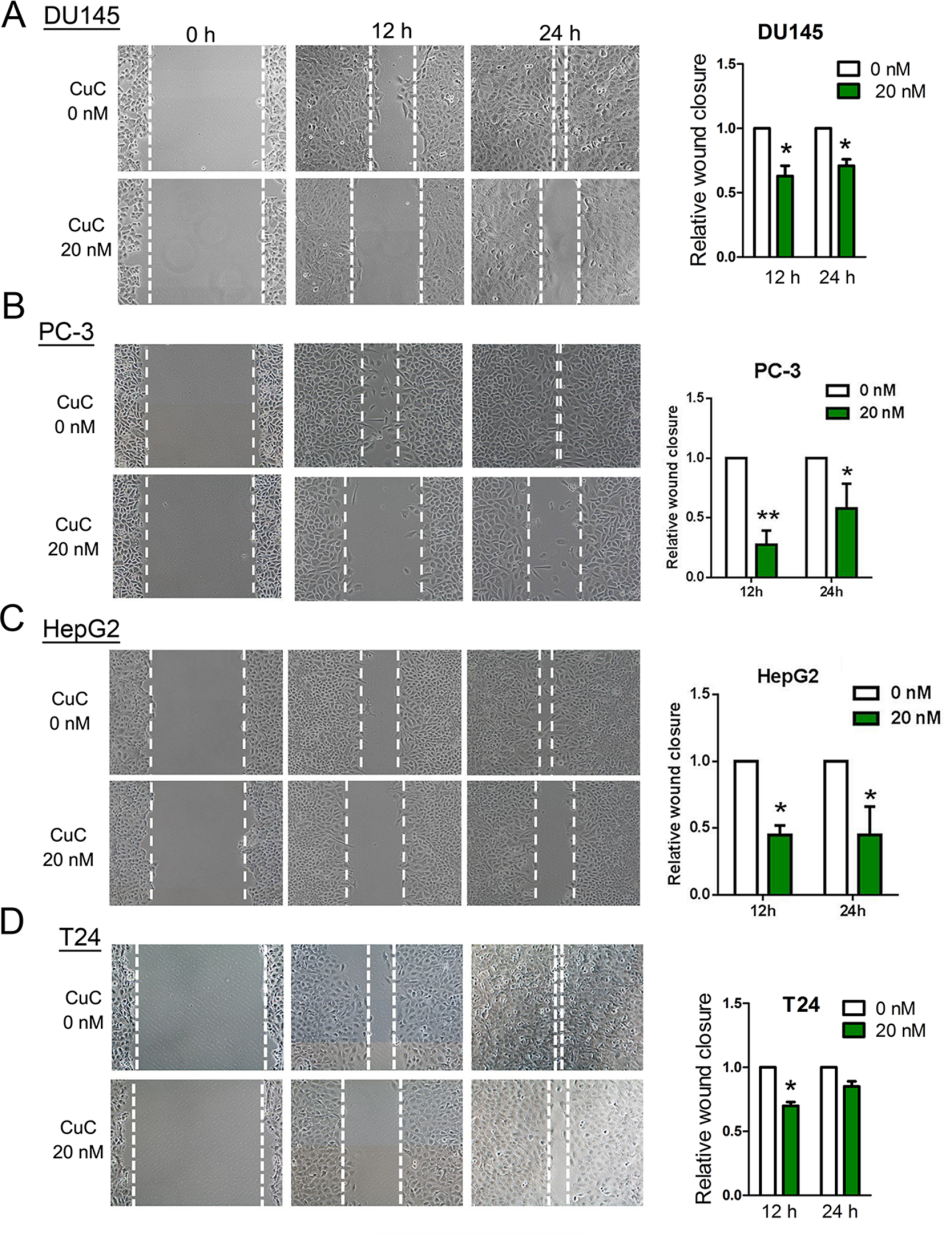
CuC inhibited cancer cell migration. Wound healing assay shows CuC inhibit the migration of DU145 **(A)**, PC-3 **(B)**, HepG2 **(C)**, and T24 **(D)** cells. Left: representative images of scratched and recovering of wounded areas (marked by white lines) on confluence monolayers of cancer cells at different time points with 20 nM CuC treatment. Right: semi-quantitative analysis of wound closure. Relative wound closure was determined by measuring the width of the wounds. Data are presented as fold changes relative to the width of the wounds made by control with no CuC present. Cells with CuC showed significantly lower migration capacity than the control cells (*P < 0.05, **P < 0.01).

To determine the functional impact of CuC treatment on cancer cell migration, we performed *in vitro* wound healing assays. Our results illustrated that a dose of CuC (20 nM) treatment effectively inhibited migration of DU145, PC-3, HepG2, and T24 cell when compared with their respective control groups at 12 and 24 h (**Figures 5A–D**).

### CuC Inhibits Cancer Cell-Derived Xenograft Tumors in Immunodeficient Mice

**Figure 6:**
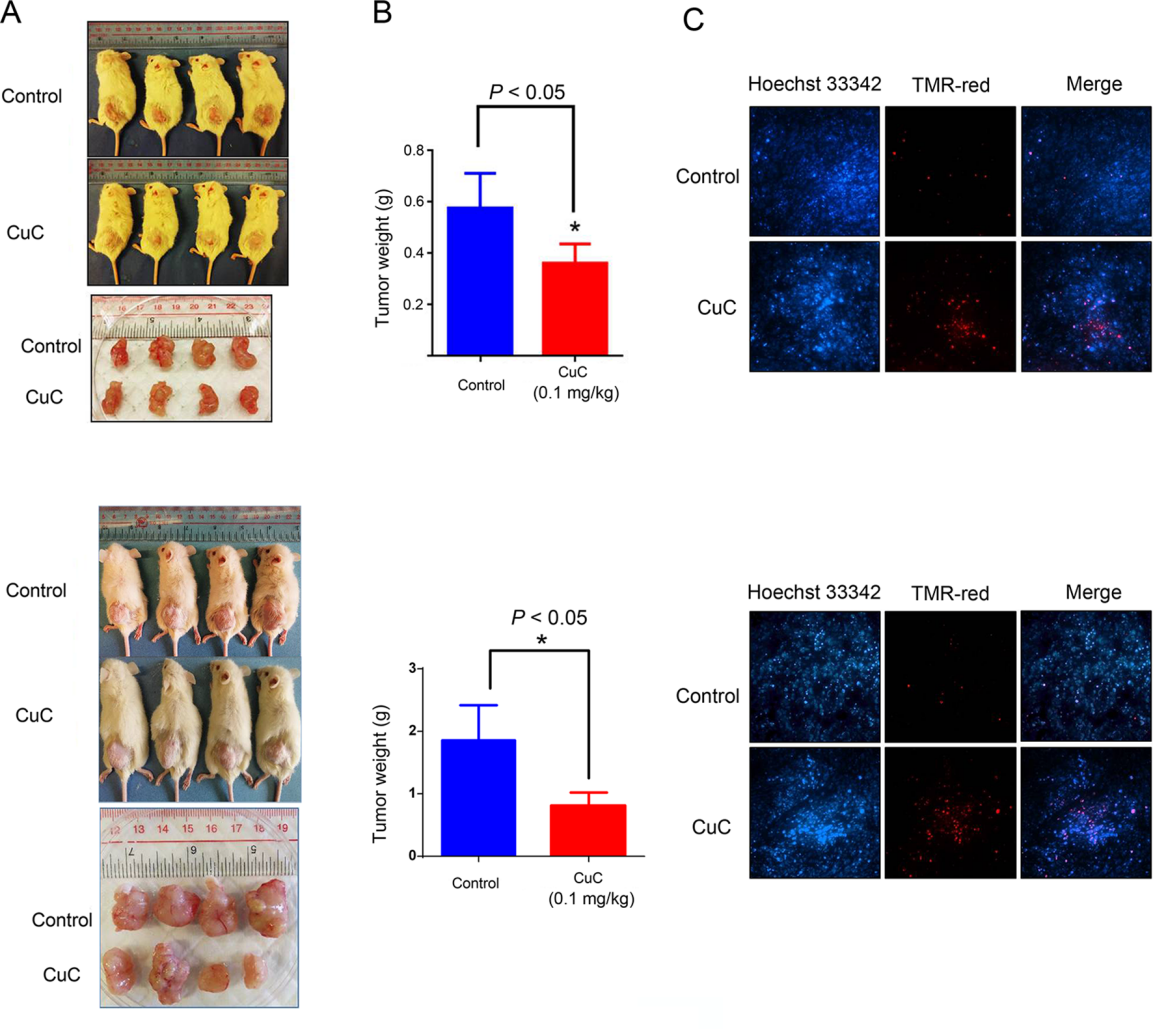
CuC inhibits growth of cancer xenograft tumors *in vivo*. Severe combined immunodeficiency (SCID) mice were inoculated subcutaneously with HepG2 or PC-3 cells. Intraperitoneal administration of CuC (0.1 mg/kg, three times per week) started after the tumor size developed to 200 mm^3^. **(A)** Representative photographs of SCID mice bearing cancer cell-derived xenograft tumors treated with vehicle control or CuC. Tumors were isolated at the end of experiment with mice sacrificed by non-painful anesthesia. Tumors were much smaller in CuC-treated mice compared with vehicle control mice. Upper panel: HepG2 xenograft; lower panel: PC-3 xenograft. **(B)** The tumor weights were measured at the end of the *in vivo* assay * *P* < 0.05. The tumor weights represent means ± SD (*n* = 4). Upper panel: HepG2 xenograft; lower panel: PC-3 xenograft. **(C)** Represented photos show TUNEL assay results of cell death in HepG2 or PC-3 xenografts. TMR-red: DNA damage; Hoechst 33342: nuclear. Results show that CuC tumors display much intense cell death signals. Upper panel: HepG2 xenograft; lower panel: PC-3 xenograft.

We further examined the *in vivo* anti-cancer effect of CuC using HepG2 and PC-3 xenograft models in SCID mice. As shown in **Figure 6A**, CuC significantly inhibited both *in vivo* tumor growth of HepG2 and PC-3 xenografts. After 28 days, the average tumor weights of HepG2 xenograft tumors were 0.58 ± 0.14 and 0.37 ± 0.09 g in the control and CuC-treated groups, respectively. For PC-3 tumors, the average weights of vehicle control were 1.81 ± 0.5 g versus CuC-treated tumors 0.75 ± 0.23 g (**Figure 6B**). No significant differences of body weight were observed between CuC-treated and control animals (data not shown), suggesting that CuC causes low host toxicity at a therapeutic dose. To determine the intratumor apoptosis caused by CuC at a molecular level, TUNEL assay was used to detect and quantify DNA breaks. Results obtained from fluorescence microscope showed that there were much intense signals of DNA cleavage in CuC-treated tumors in both HepG2 and PC-3 xenografts than vehicle control tumors (**Figure 6C**).

### CuC Altered Gene Expressions and Akt Pathway

**Figure 7:**
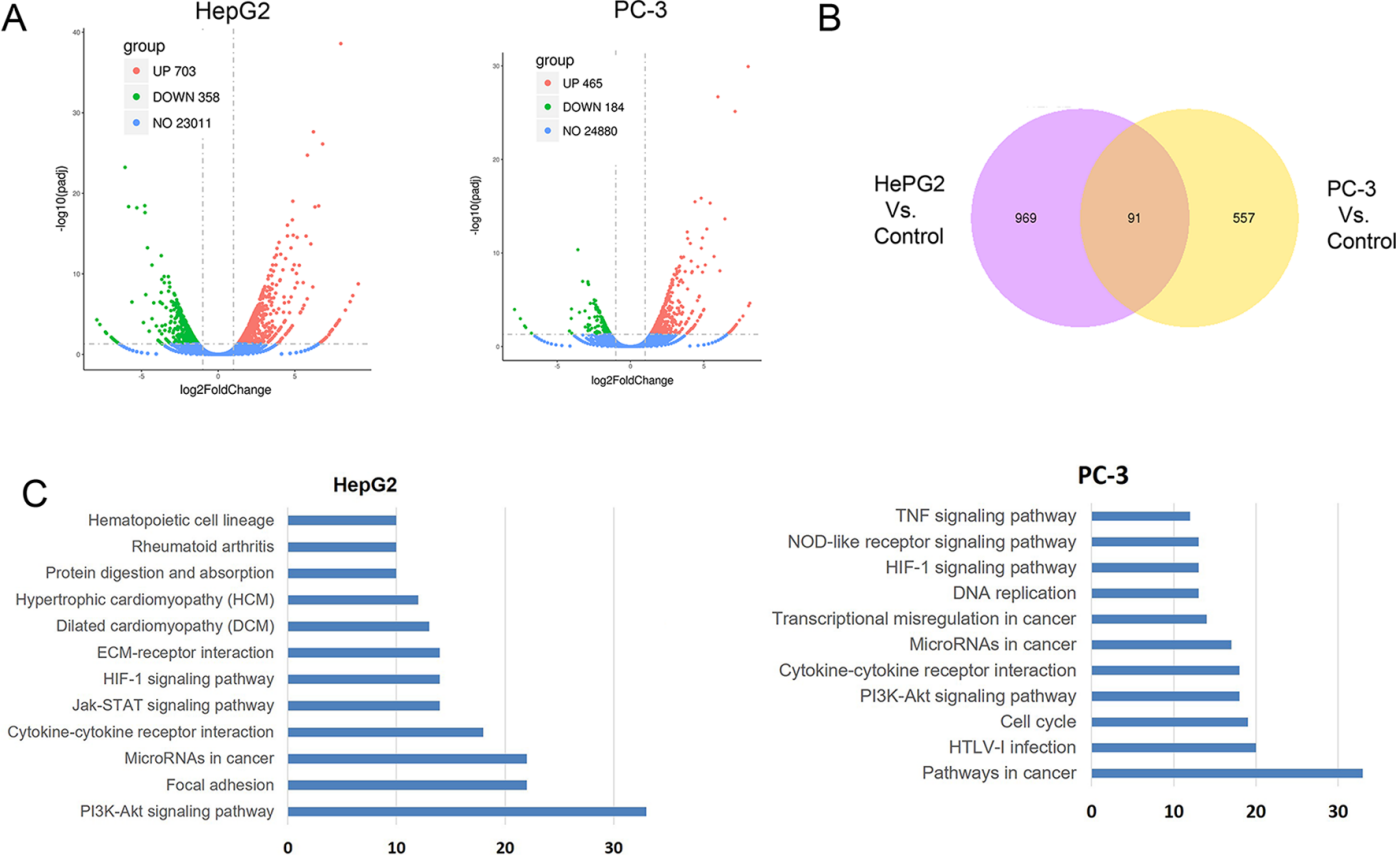
CuC altered genes in HepG2 and PC-3 cells by RNA-Seq transcriptional profiling. **(A)** Differentially expressed genes in HepG2 and PC-3 cell response to CuC treatment. In HepG2 cells, there are 703 genes up-regulated and 358 genes down-regulated upon CuC treatment compared with control cells. For PC-3 cells, 465 genes show up-regulation and 184 genes shows down-regulation. **(B)** 91 genes affected by CuC were found in both HepG2 and PC-3 cells. **(C)** Gene Ontology analysis of CuC-responsive genes in HepG2 and PC-3 cells. PI3K-Akt signaling pathway, microRNAs, and cytokine–cytokine receptor interaction pathways are both significantly changed in both two cells lines.

To further understand the molecular mechanism underlying the effect of CuC on cancer cells, we surveyed the gene expression alterations and signaling pathways that affected by CuC treatment using RNA-Sequencing. It shows that CuC altered the expression of 1,061 genes in HepG2 cells, with 703 genes up-regulated and 358 down-regulated. In PC-3, there were 649 genes changed, with 465 up-regulation and 184 down-regulation (**Figure 7A**). When we compared HepG2 with PC-3, there were 91 genes showing alternations in both lines affected by CuC (**Figure 7B**). GO and KEGG pathway analysis of these altered genes pointed to the possible targets and pathways of CuC. Results show that PI3K-Akt, focal adhesion, microRNAs in cancer, cytokine–cytokine receptor interaction, and Jak-STAT signaling pathway are the most affected pathways by CuC in HepG2 cells, whereas in PC-3 cells, microRNAs, cytokine–cytokine receptor interaction, and PI3K-Akt signaling pathways were also changed. Moreover, genes belong to pathways in cancer, HTLV-1 infection and cell cycle pathways were also disturbed in PC-3 by CuC (**Figure 7C**). For PI3K-Akt pathway, FOXO3, CSF1, PPP2R2B, DDIT4, PHLPP2, ITGA2, ITGB4, Casp9, and CDKN1A (p21) were up-regulated, while CHAD, IL2RG, JAK3, CREB3L1, MCL1, and CCND3 showed down-regulation. Cyclin genes such as CCND1 (cyclinD1) and CCND3 (cyclin D3) were significantly affected (**Supplementary Figure 1A**). The expression of Akt downstream genes was also examined by Q-PCR, and the results showed that CDKN1A (p21), CDKN1B (p27), and FOXO were significantly up-regulated by low-concentration CuC treatment (0.01–0.1 μM), which was consistent with the RNA-Seq data. Moreover, the Akt downstream genes GSK3A, GSK3B, and CHUK genes were also found to be up-regulated by CuC (0.01–0.1 μM) (**Supplementary Figure 1B**). Together, these RNA-Seq and Q-PCR data indicated that CuC altered multiple signaling pathways in cancer cells, and PI3K-Akt pathway was majorly affected.

### CuC Inhibits Akt Phosphorylation

**Figure 8:**
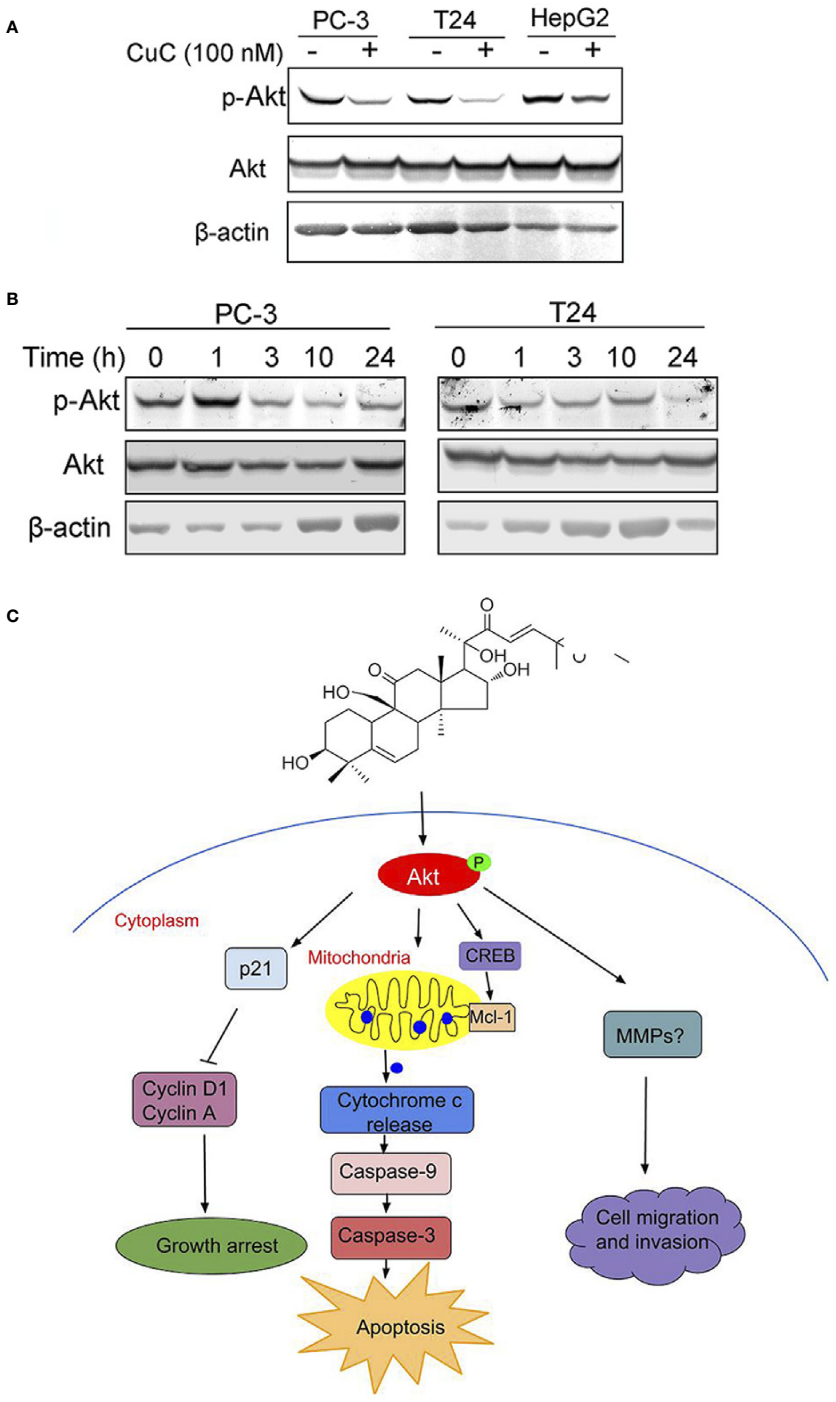
Molecular working model of CuC in cancer cells. **(A)** Western blot (WB) analysis of p-Akt and Akt in PC-3, T24, and HepG2 cells with or without CuC. p-Akt (Ser473) was significantly decreased in the presence of 100 nM CuC. Cells were treated for 24 h. **(B)** p-Akt (Ser473) and Akt expression in PC-3 and T24 cells under CuC (100 nM) treatment at different time points (0, 1, 3, 10, and 24 h). **(C)** Schematic diagram illustrated the bio-relevant context of antitumor activities of CuC, and the proposed cell growth arrest, apoptosis, and cell migration inhibition are illustrated.

The Akt pathway cascade is the most frequently dysregulated signaling pathway in human cancers, and Akt protein is overexpressed or excessively activated in a variety of cancers. High expression of activated Akt is closely related to cancer progression and can provide major cell survival signal in many cancer types (Nitulescu et al., 2016). Our RNA-Seq analysis data suggested that PI3K-Akt pathway was majorly involved in CuC-induced cellular events. Thus, we were interested to further investigate the specific effect of CuC on Akt signaling. Western blot results revealed CuC treatment inhibited phosphorylation of Akt at Ser473 in LNCaP, T24, and HepG2 cancer cells, while it showed no significant change of pan-Akt (**Figure 8A**). To determine the effect of CuC on inhibition of p-Akt over time course, cancer cells were treated with 100 nM CuC for 1–24 h. For prostate cancer PC-3 cells, the effect of CuC on inhibition of p-Akt (Ser473) started at 3 h and most prominently at 24 h post incubation. For bladder cancer cell T24, p-Akt inhibition effect was even faster, which began at 1 h post incubation and reached the lowest point at 24 h (**Figure 8B**), suggesting that CuC effectively inhibited the Akt signaling in prostate cancer, bladder cancer, and liver cancer cells, and the blocking of Akt phosphorylation at Ser473 was in a time-dependent manner. Moreover, down-regulated expression of p-Akt was observed in PC-3 xenograft tumors detected by immunohistochemistry (**Supplementary Figure 3**). These results suggested that inhibition of Akt pathway was an associated event with CuC-induced growth arrest and apoptosis in cancer cells.

## Discussion

Cancer is a disease wherein cancer cells abnormally proliferate and differentiate, resulting in the second most common cause of death worldwide. Chemotherapy that induces apoptosis and cell growth arrest and inhibits angiogenesis, cell metabolism, and invasion remains the one of the major cancer treatment strategies. Remarkably, natural products are the most important anti-cancer agents. Actually, 75% anti-tumor compounds used in medicine to combat human cancer are natural product resources. CuC is a natural product that confers the bitter taste in cucumber. The signaling pathways and genes that control its biosynthesis were dissected recently (Shang et al., 2014). Our results identified, for the first time, the anti-cancer effects of CuC in cancer cells. These results revealed that CuC significantly inhibited cancer cell growth by induction of cell cycle arrest, apoptosis, and inhibition of cell migration, which are consistent with the anti-cancer properties of other types of cucurbitacins (Cai et al., 2015). IC50s of CuC were identified in the nanomolar range (9.6 to 158.7 nM), which is much lower than those of other cucurbitacin analogues, the IC50 of which is usually 0.1 to 1 μM (Cai et al., 2015). These results increased the potency of CuC as an effective anti-cancer candidate.

Agents that induce cell cycle arrest and apoptosis could be potential therapeutic drugs for cancer therapy. Cucurbitacins have been demonstrated to induce cell growth arrest and apoptosis in various cancer cells. Our results demonstrated that CuC is also a cell cycle arrest- and apoptosis-inducing agent. However, it seems that CuC caused cell cycle arrest at different stages in different cancer cells (G1 stage of DU145 and LNCaP cells but G2/M arrest in T24, HepG2, and PC-3 cells). G1 arrest prevents DNA repair of cancer cells and inhibits them from entering S phase, which is mediated by cyclins and cyclin-dependent kinases (CDKs). Our results show that cyclin D1, which plays critical roles in G1/S transition, was significantly decreased by CuC in LNCaP, which would mediate the G1 arrest in these cells. However, in CuC-treated T24 and HepG2 cells, in which G2/M cycle was abrogated, CDK inhibitor p21 was significantly up-regulated in a p53-independent way (**Figure 3C**). These results suggest that CuC inhibits growth of different cancer cells through induction of G1/S or G2/M phase of cell cycle arrest *via* modulating different cell cycle regulatory proteins.

Akt/protein kinase B (PKB) is known to be the key mediator that transduces signals from activated growth factors and oncogenes to downstream targets that control human malignancy development. Aberrant Akt expression and hyperactivation of Akt, which was commonly occurred in human cancers *via* mechanisms including loss of PTEN, mutation or amplification of phosphatidylinositol 3-kinase (PI3K), activation or mutation of receptor kinases, and oncogenes that affect upstream modulators of Akt, therefore, promote cancer cell survival and proliferation. The overexpression and activation of Akt are often associated with chemo-resistance or radiotherapy resistance (Katsogiannou et al., 2015). Therefore, Akt has been considered as an attractive target for cancer therapy. Natural products, such as curcumin, selenium, and the flavonoid apigenin, has been demonstrated to suppress tumor growth *in vitro* and *in vivo* by inhibiting Akt activity (Crowell et al., 2007). Our results showed that CuC inhibited Akt phosphorylation at one of its key regulatory sites, Ser473, without affecting pan-Akt expression. The attenuation of Akt signaling then may impair the activation of its substrates and downstream targets that are critical members involved in the progression of cell cycle, cell survival, metabolism, and DNA damage (Carnero and Paramio, 2014). In the present study, RNA-Seq analysis showed that downstream of Akt pathway including cell cycle regulators p21/cyclins, FOXOs, and cell survival and apoptosis mediators CREB/Mcl-1 signaling were affected by CuC (**Supplementary Figures 1A, B**). These changes were confirmed by their protein levels of p21 accumulation and decrease of cyclin A and D1, as well as the increase of cleaved caspase-3 and cleaved caspase-9. Based on these, we speculated that CuC induces growth arrest and apoptosis of cancer cells is associated event with the attenuation of Akt phosphorylation, which then mediated the change of its downstream targets of cell cycle and apoptosis regulators. However, the specific function and involvement of upstream of Akt, such as cytokines and Akt phosphorylation modulators PP2A (Seshacharyulu et al., 2013) and PHLPP (O’Neill et al., 2013), and the downstream of Akt, such as CREB/Mcl-1 signaling (Milot and Filep, 2011), which significantly affected by CuC treatment as shown in RNA-Seq data, need further investigation.

## Conclusions

In conclusion, our study indicates for the first time that CuC displays multiple anti-cancer activities *in vitro* and *in vivo via* attenuating key oncogenic signaling pathways. Specifically, CuC induced cancer cell growth arrest, apoptosis, and cell migration inhibition *via* suppression of Akt phosphorylation, followed by modulation of p21/cyclin signals, mitochondria-dominated caspase pathways, as well as signaling involved in cancer cell migration and invasion, as illustrated by the molecular working model proposed in **Figure 8C**.

## Data Availability Statement

The raw data supporting the conclusions of this manuscript will be made available by the authors, without undue reservation, to any qualified researcher. The RNA sequencing data has been uploaded to the CEO database, the accession number is GSE138367.

## Ethics Statement

This study was carried out in accordance with the recommendations of Experimental Animal Welfare Ethics Guidelines, Animal Experimentation Ethics Committee, Southern Medical University. The protocol was approved by the Animal Experimentation Ethics Committee, Southern Medical University.

## Author Contributions

DW, XL, and WH conceived and designed the research. ZW, ML, and JZ developed the methodology. DW, ZW, ML, and FW (5th author) acquired the data. YS, FW (7th author) and XZ analyzed and interpreted the data. XL and WH supervised the study. All authors approved the submission of the manuscript.

## Funding

This work was supported by the Science and Technology Project of Shenzhen (JCYJ20170307144115825 and JCYJ20180508163203807), Shenzhen Key Laboratory of Viral Oncology (ZDSYS201707311140430), National Natural Science Foundation of China (project numbers 81772737 and 81502570), National Science Foundation Projects of Guangdong Province (2017B030301015), and Sanming Project of Medicine in Shenzhen (SZSM201612023 and SZSM201412018).

## Conflict of Interest

The authors declare that the research was conducted in the absence of any commercial or financial relationships that could be construed as a potential conflict of interest.

## Abbreviations

CuC, cucurbitacin C; ATCC, American Type Culture Collection; MTT, 3-(4,5-dimethylthiazole-2-yl)-2,5-biphenyl tetrazolium bromide; SD, standard deviation; ECL, enhanced chemi-luminescence; IC50, the half maximal inhibitory concentration; CDKs, cyclin-dependent kinases; PKB, protein kinase B; PI3K, phosphatidylinositol 3-kinase.
