# Corrigendum: *In vitro* and *in vivo* antitumor activity of cucurbitacin C, a novel natural product from cucumber

**DOI:** 10.3389/fphar.2024.1334971

**Published:** 2024-03-04

**Authors:** Dinglan Wu, Zhu Wang, Muqi Lin, Yi Shang, Fei Wang, JiaYi Zhou, Fei Wang, Xiantong Zhang, Xiaomin Luo, Weiren Huang

**Affiliations:** ^1^ Shenzhen Key Laboratory of Viral Oncology, The Clinical Innovation & Research Centre, Shenzhen Hospital, Southern Medical University, Shenzhen, China; ^2^ Department of Urology, People’s Hospital of Longhua Shenzhen, Southern Medical University, Shenzhen, China; ^3^ School of Pharmaceutical Sciences, Health Science Center, Shenzhen University, Shenzhen, China; ^4^ Agricultural Genome Institute at Shenzhen, Chinese Academy of Agricultural Science, Shenzhen, China; ^5^ Department of Urology, The Hospital of Hainan Province, Haikou, China; ^6^ Department of Urology, Shenzhen Second People’s Hospital, The First Affiliated Hospital of Shenzhen University, International Cancer Center, Shenzhen University School of Medicine, Shenzhen, China

**Keywords:** cucurbitacin C, natural product, anti-cancer, growth arrest, apoptosis, Akt pathway

In the published article, there was an error in Figures 4, 8 as published. The incorrect images were erroneously inserted.

**FIGURE 4:**
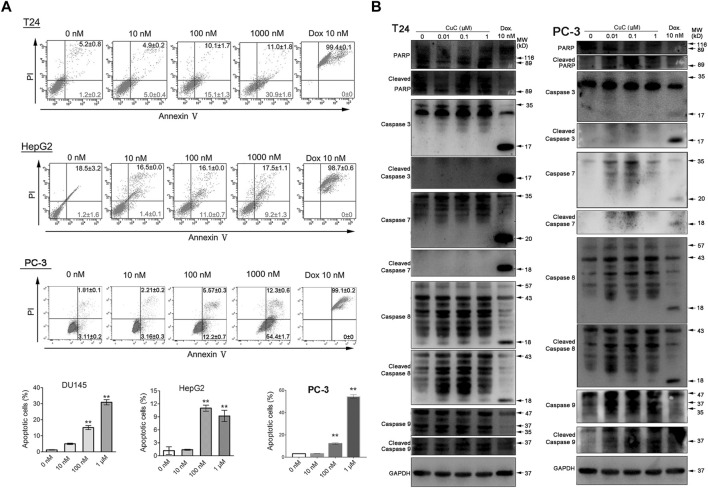
CuC induces cell apoptosis. **(A)** Apoptosis assay of cancer cells with CuC treatment by annexin V-FITC and PI staining. CuC induced significant early apoptosis at 100 mM and 1 µM treatment in T24, HepG2 and PC-3 cells (**, *p* < 0.01). **(B)** Western blot (WB) analysis of indicated apoptotic markers after 48 h dosage of serial CuC in T24 and PC-3 cells.

**FIGURE 8:**
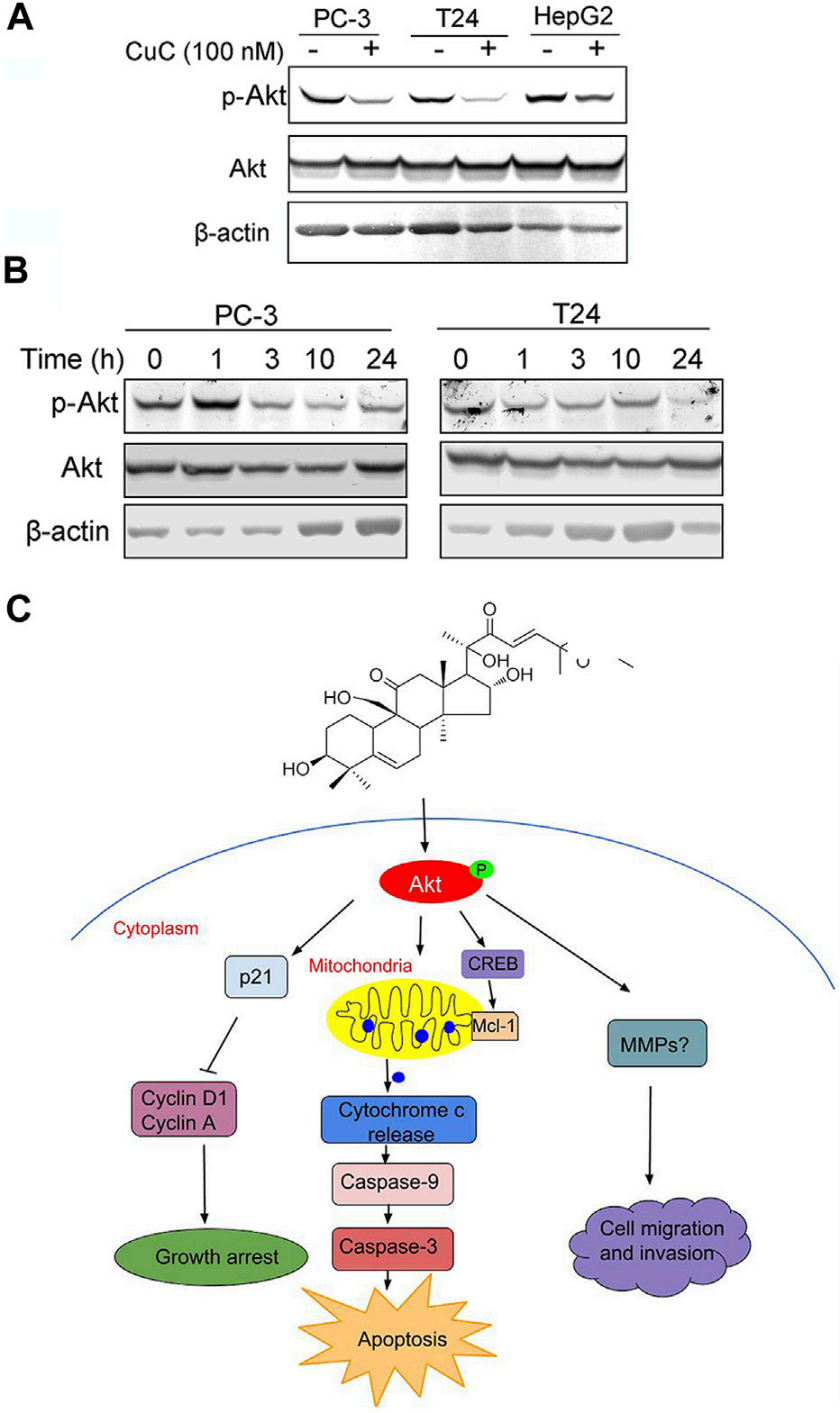
Molecular working model of CuC in cancer cells. **(A)** Western blot (WB) analysis of p-Akt and Akt in PC-3, T24, and HepG2 cells with or without CuC. p-Akt (Ser473) was significantly decreased in the presence of 100 nM CuC. Cells were treated for 24 h **(B)** p-Akt (Ser473) and Akt expression in PC-3 and T24 cells under CuC (100 nM) treatment at different time points (0, 1, 3, 10, and 24 h). **(C)** Schematic diagram illustrated the bio-relevant context of antitumor activities of CuC, and the proposed cell growth arrest, apoptosis, and cell migration inhibition are illustrated.

Specifically, in Figure 4B, the blot of Caspase 3 of PC-3 cell was inadvertently displayed as the cleaved caspase-3. In Figure 8A, the blot of β-actin image was mistakenly showed, due to the carelessness of the picture combination and image processing. Additionally, there is an error in the caption of Figure 8A. The published legend states: “Western blot (WB) analysis of p-Akt and Akt in PC-3, T24, and LNCaP cells with or without CuC”. The legend of Figure 8A is corrected as: “Western blot (WB) analysis of p-Akt and Akt in PC-3, T24, and HepG2 cells with or without CuC.” Finally, in the caption of Figure 4A, “T-24” is corrected as “T24.”

The corrected Figures 4, 8 and their captions appear below.

The authors apologize for these errors and state that this does not change the scientific conclusions of the article in any way. The original article has been updated.

