# Supplementary figures and images for: In Vitro and In Vivo Antitumor Activity of Cucurbitacin C, a Novel Natural Product From Cucumber
Source: Front Pharmacol. 2019 Nov 8;10:1287. doi: 10.3389/fphar.2019.01287 (PMC6857091; doi:10.3389/fphar.2019.01287)

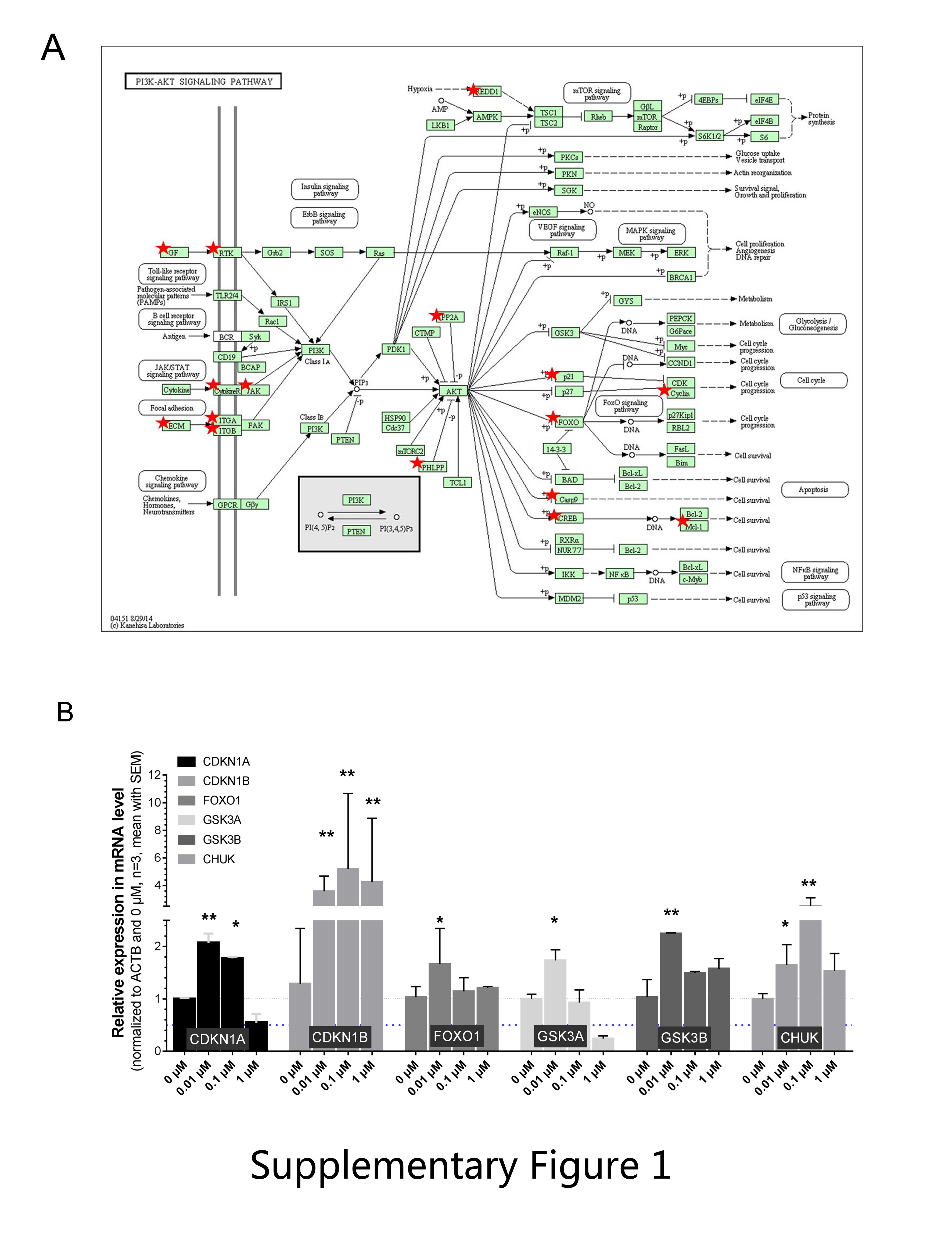

Supplement: Supplementary file 1 [file Image_1.tif]

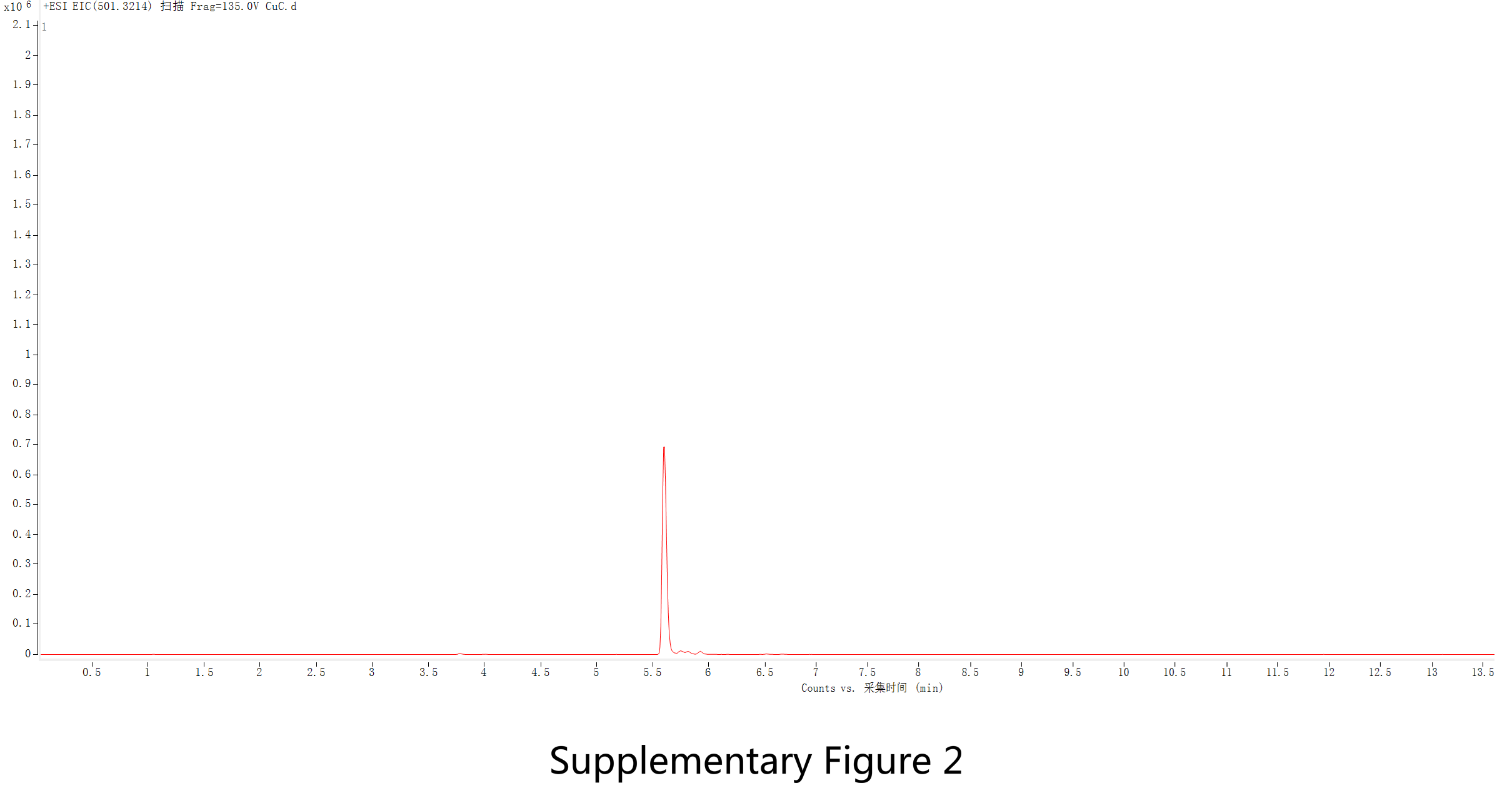

Supplement: Supplementary file 2 [file Image_2.tif]

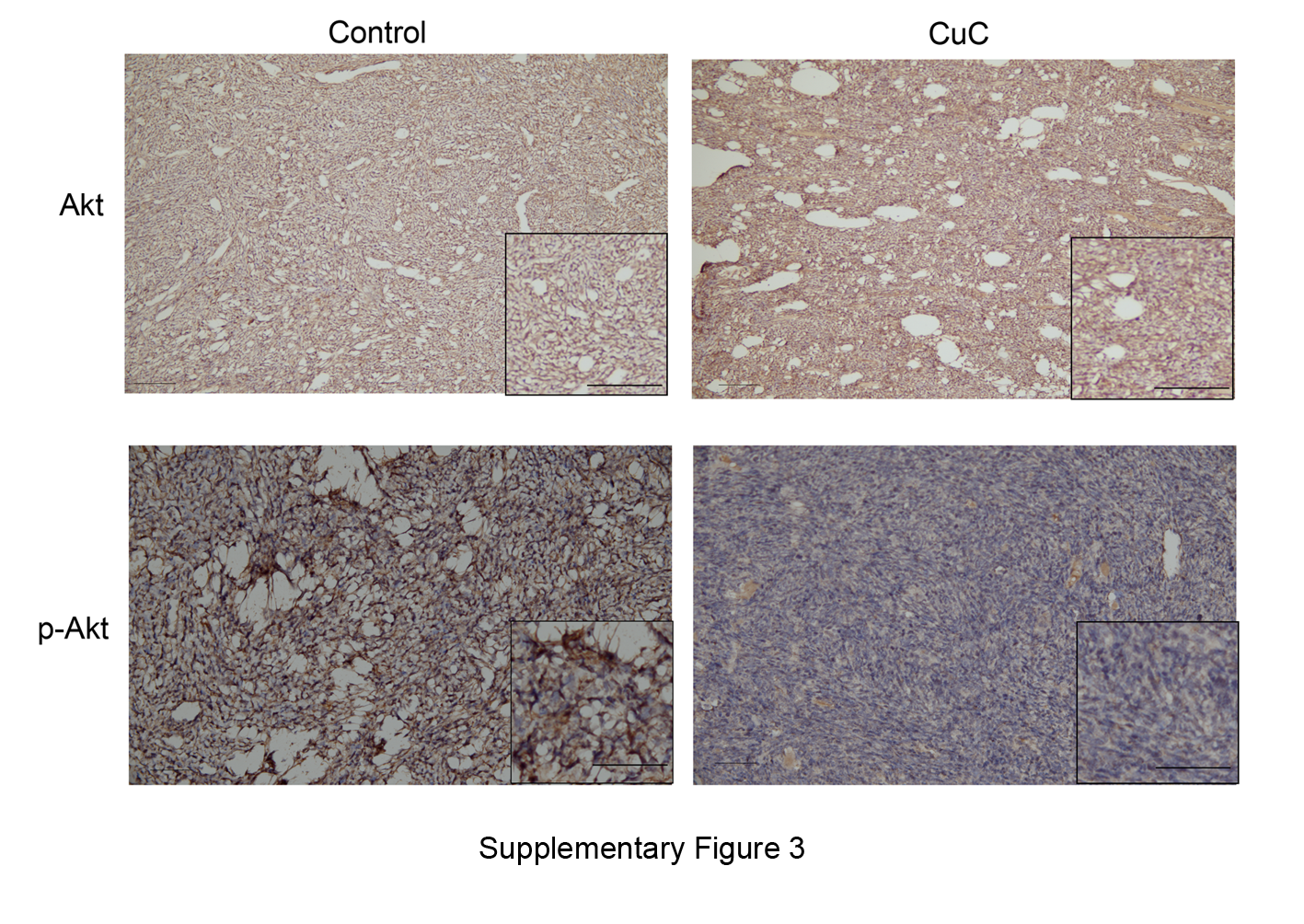

Supplement: Supplementary file 3 [file Image_3.tif]
